# Supplementary material for: Altered Metabolism in Knockdown Lines of Two HXXXD/BAHD Acyltransferases During Wound Healing in Potato Tubers
Source: Plants (Basel). 2024 Oct 26;13(21):2995. doi: 10.3390/plants13212995 (PMC11548462; doi:10.3390/plants13212995)
Supplement: Supplementary file 1 [file plants-13-02995-s001.zip › plants-3257296-supplementary.pdf]

Altered metabolism in knockdown lines of two HXXXD/BAHD acyltransferases during wound healing in potato tubers

Jessica L. Sinka [1†], Indira Castillo-Queralta [1†], Lorena S. Yeung [1], Isabel Molina [2], Sangeeta Dhaubhadel [3] and Mark A. Bernards [1,*]

Supplemental Data

Table S1 **Primers used for PCR amplification, genotype analyses and qRT-PCR.** The underline base pairs represent the *attB1*(in the forward primers) and *attB2* (in the reverse primers) recombinant sequences for Gateway® cloning. Asterisks (*) indicate the references genes used for data normalization based on Nicot *et al.* (2005) [34].

**Gene Name**	**Accession #**	**Primer Name**	**Sequence (5'-3')**	**Amplicon size (bp)**	**Primer Efficiency (%)**
PCR Amplification					
StFHT	PGSC0003DMG400031731	StFHT-RNAi-F<br>StFHT-RNAi-R	GGGGACAAGTTTGTACAAAAAAGCAGGCTTCAATTCTTGGGGTGAAACTGCT<br>GGGGACCACTTTGTACAAGAAAGCTGGGTCGTAATGCCTGAGTTCGCGCTT	304	-
StHCT	PGSC0003DMG400014152	StHCT-RNAi-F<br>StHCT-RNAi-R	GGGGACAAGTTTGTACAAAAAAGCAGGCTTCAACGAGTGTGTCCATCTTCAA<br>GGGGACCACTTTGTACAAGAAAGCTGGGTCCTTAAATAATCGTTGTCCATT	351	-
St430	PGSC0003DMG400007171	St430-RNAi-F<br>St430-RNAi-R	GGGGACAAGTTTGTACAAAAAAGCAGGCTTCAACGGACCAATGTTGCACTCT<br>GGGGACCACTTTGTACAAGAAAGCTGGGTCATCCATCATCGTTCTCACCTT	322	-
Genotyping Primers					
pK7GWIWG2D(II),0	RNAi Vector	pK7GWIWG2-F<br>pK7GWIWG2-R	TCAAGCTGACCTGCAAACAC<br>GGGCGAAGAAGTTGTCCATA	796	-
StFHT	PGSC0003DMG400031731	StFHT-gDNA-F<br>StFHT-gDNA-R	AATTCTTGGGGTGAAACTGCT<br>GTAATGCCTGAGTTCGCGCTT	304	-
StHCT	PGSC0003DMG400014152	StHCT-gDNA-F<br>StHCT-gDNA-R	AACGAGTGTGTCCATCTTCAA<br>AAAATAGCCTGGTGGGAGAGA	229	-

St430	PGSC0003DMG400007171	St430-gDNA-F<br>St430-gDNA-R	AACGGACCAATGTTGCACTCT<br>ATCCATCATCGTTCTCACCTT	332	-
qRT-PCR primers					
StFHT	PGSC0003DMG400031731	StFHT-qRT-F<br>StFHT-qRT-R	TGTGAAGCAAGGAGTGCCAA<br>ACCGGCACGGCTATATTCTG	99	103.7
StHCT	PGSC0003DMG400014152	StHCT-qRT-F<br>StHCT-qRT-R	ATGGCTCGTGGTTTGGACAT<br>ACTGAGGCTGAGGTGGATCA	82	110.5
St430	PGSC0003DMG400007171	St430-qRT-F<br>St430-qRT-R	ACGATGAGGGAGCGCCTTTT<br>TGACTTGGACAA	165	93.1
*StEF1-α**	PGSC0003DMG400023272	StEF1a-qRT-F<br>StEF1a-qRT-R	TGGTCGTGTTGAGACTGGTG<br>AACATTGTCACCGGGGAGTG	133	108.1
*StAPRT**	PGSC0003DMG4	StAPRT-qRT-F<br>StAPRT-qRT-R	GAACCGGAGCAGGTGAAGAA<br>GAAGCAATCCCAGCGATACG	121	104.0

Table S2 **Medium composition for *Agrobacterium* infection, callus induction, shoot induction, and root induction.** All culture media were adjusted to pH 5.8. AIM (Agrobacterium infection medium), CIM (callus induction medium), SIM (shoot induction medium), RIM (root induction medium).

**Media components** (per 1 L medium)	**Type of medium** AIM	CIM	SIM	RIM
MS salts (g)	4.4	4.4	4.4	4.4
MS vitamins (1000x) (mL)	1	1	1	1
Sucrose (g)	30	20	20	20
Myoinositol (mg)	0	100	100	100
IAA (mg)	0	1	1	1
Zeatin (mg)	0	2.5	2	0
GA_3 (mg)	0	0.02	0.02	0
Gelrite (g)	0	2	2	2

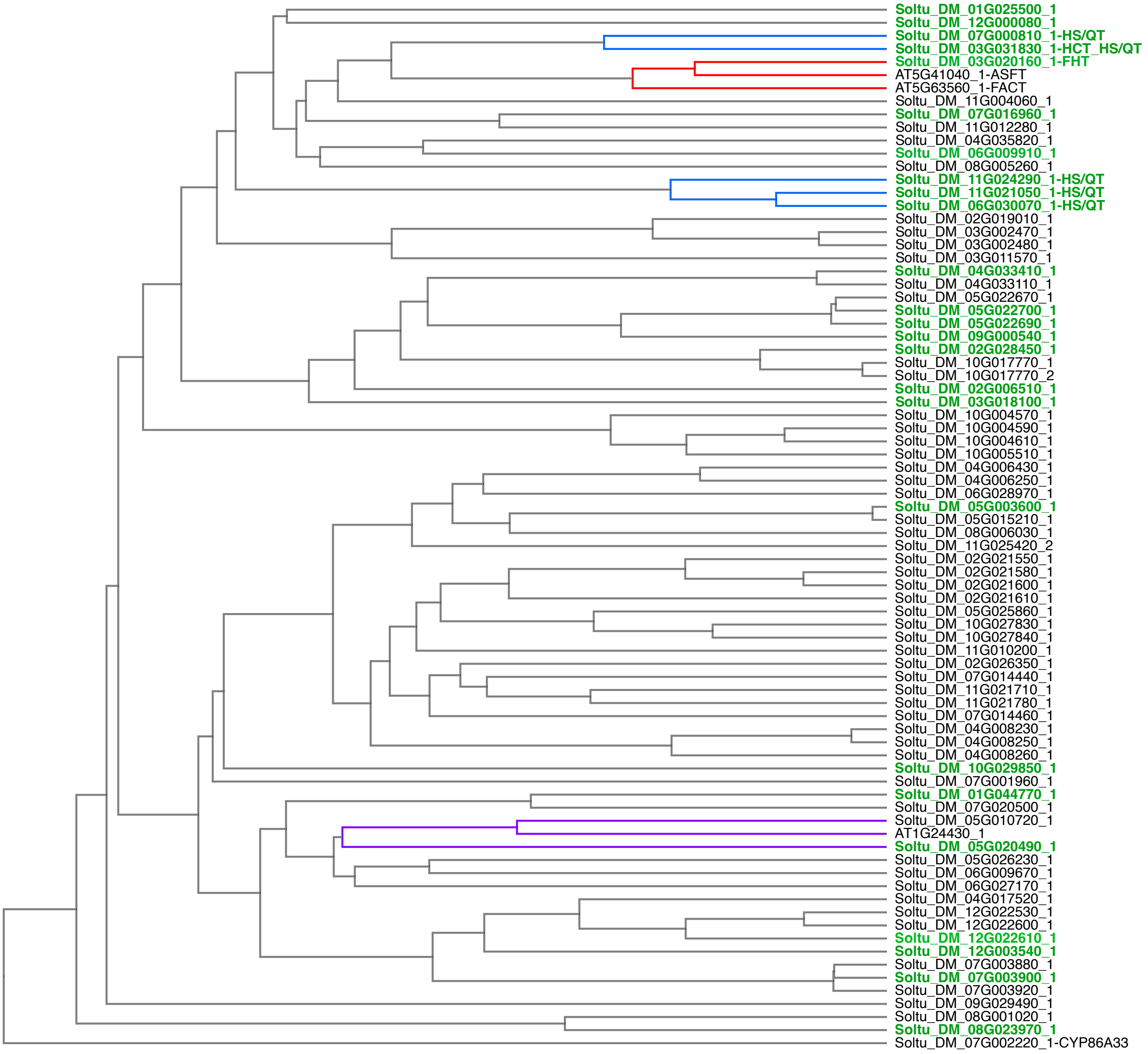

Figure S1 Potato HXXXD/BAHD Phylogeny

Seventy-five non-redundant potato HXXXD/BADH acyltransferase genes were used to create a potato HXXXD/BAHD acyltransferase neighbour-joining phylogenetic tree (1000 bootstrap) using MAFFT (mafft.cbrc.jp) based on amino acid sequences (retrieved from phytozome-next.jgi.doe.gov). Amino acid sequences for three Arabidopsis HXXXD/BAHD genes (*At5g41040, At5g63560* and *At1g24430*) were also included. StCYP86A33 (Soltu.DM.07G002220.1) was used as an outlier to root the tree. The rooted phylogenetic tree was rendered using Phylo.IO (http://phylo.io/; Robinson *et al.* (2016)). Protein annotations for the 28 potato HXXXD/BADH acyltransferase genes induced by wounding (Woolfson et al. 2022) are highlighted in green. Two distinct HS/QT subclades are shown in blue, while the StFHT subclade is shown in red. A distinct subclade including the putative At1g24430 homolog Soltu_DM_05G020490 is shown in purple.

A

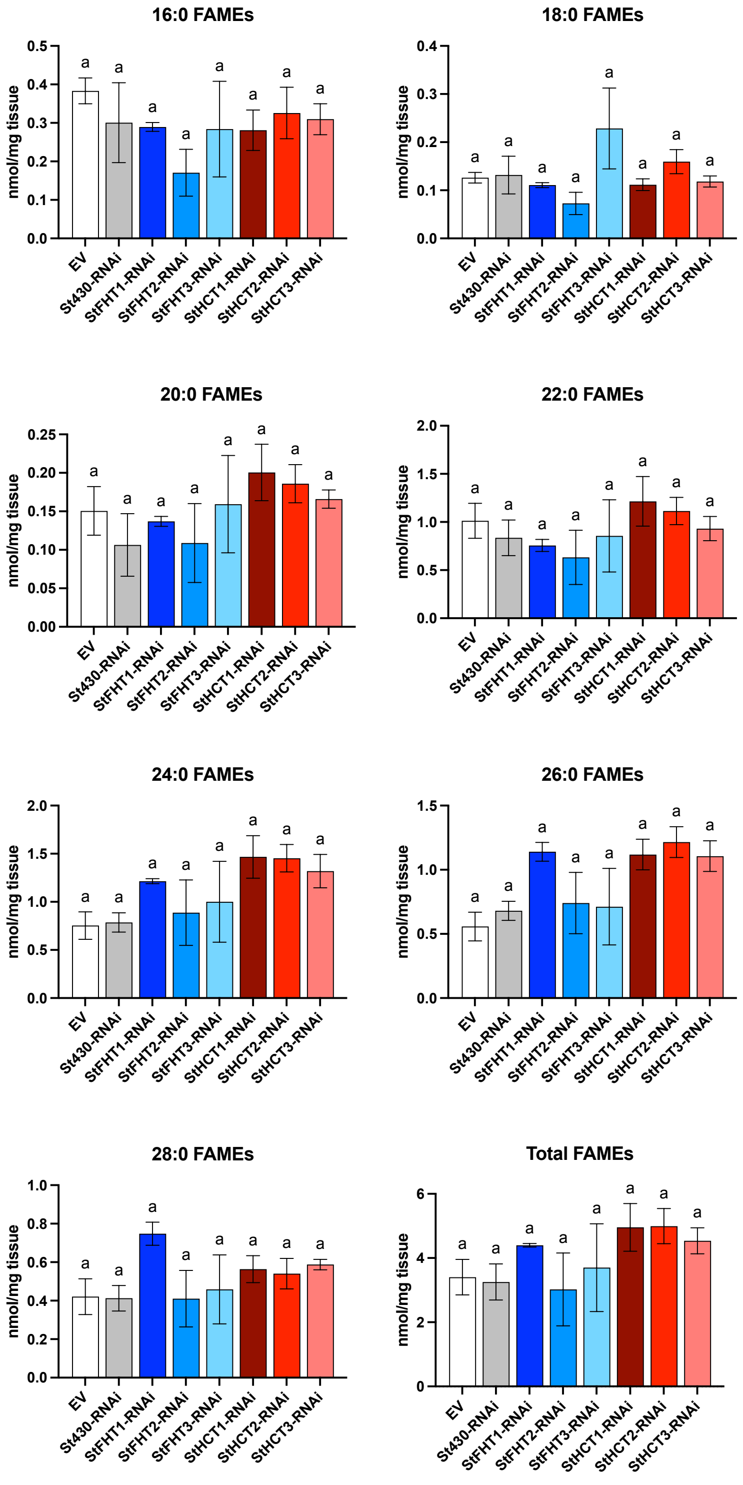
16:0 FAMEs
18:0 FAMEs
20:0 FAMEs
22:0 FAMEs
24:0 FAMEs
26:0 FAMEs
28:0 FAMEs
Total FAMEs
nmol/mg tissue
EV
St430-RNAi
StFHT1-RNAi
StFHT2-RNAi
StFHT3-RNAi
StHCT1-RNAi
StHCT2-RNAi
StHCT3-RNAi

B

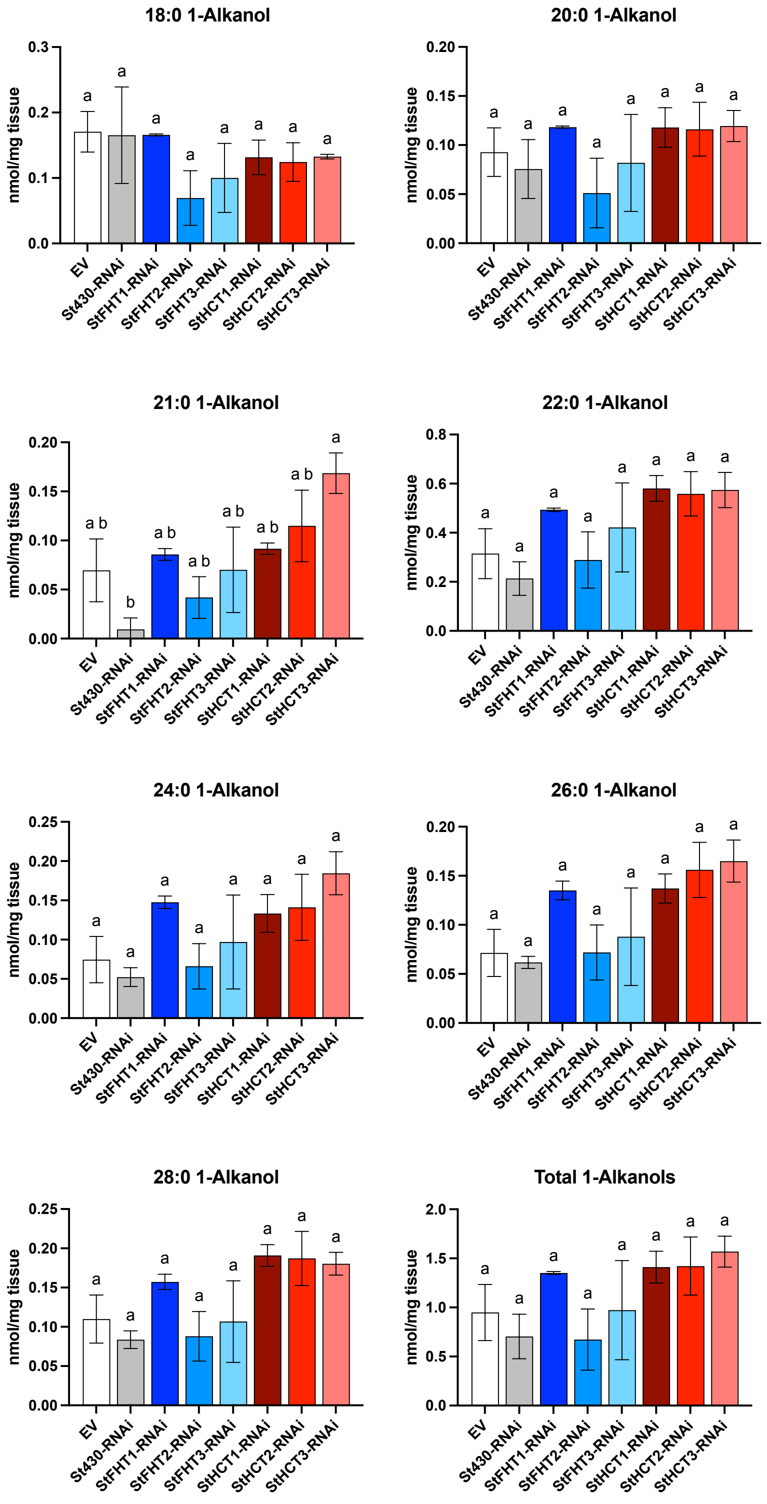

18:0 1-Alkanol
20:0 1-Alkanol
21:0 1-Alkanol
22:0 1-Alkanol
24:0 1-Alkanol
26:0 1-Alkanol
28:0 1-Alkanol
Total 1-Alkanols
nmol/mg tissue
EV
St430-RNAi
StFHT1-RNAi
StFHT2-RNAi
StFHT3-RNAi
StHCT1-RNAi
StHCT2-RNAi
StHCT3-RNAi

C

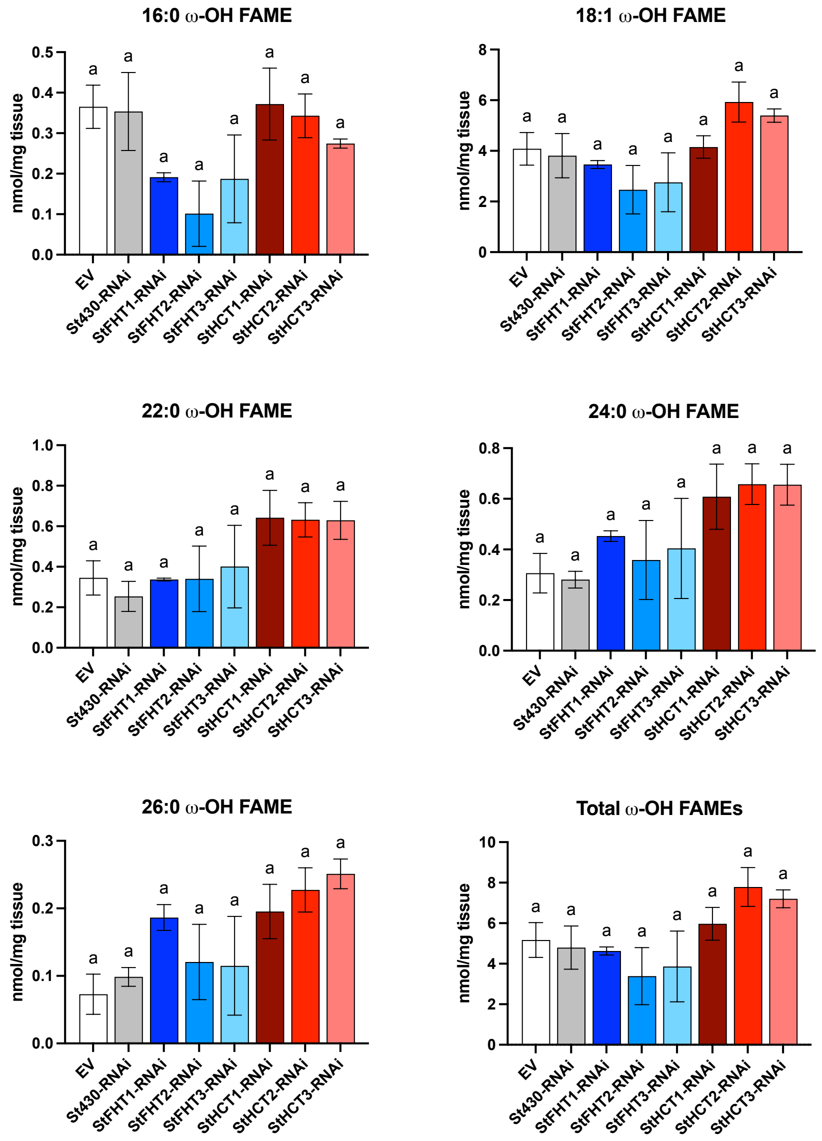

16:0 ω-OH FAME
18:1 ω-OH FAME
22:0 ω-OH FAME
24:0 ω-OH FAME
26:0 ω-OH FAME
Total ω-OH FAMEs
nmol/mg tissue
EV
St430-RNAi
StFHT1-RNAi
StFHT2-RNAi
StFHT3-RNAi
StHCT1-RNAi
StHCT2-RNAi
StHCT3-RNAi

D

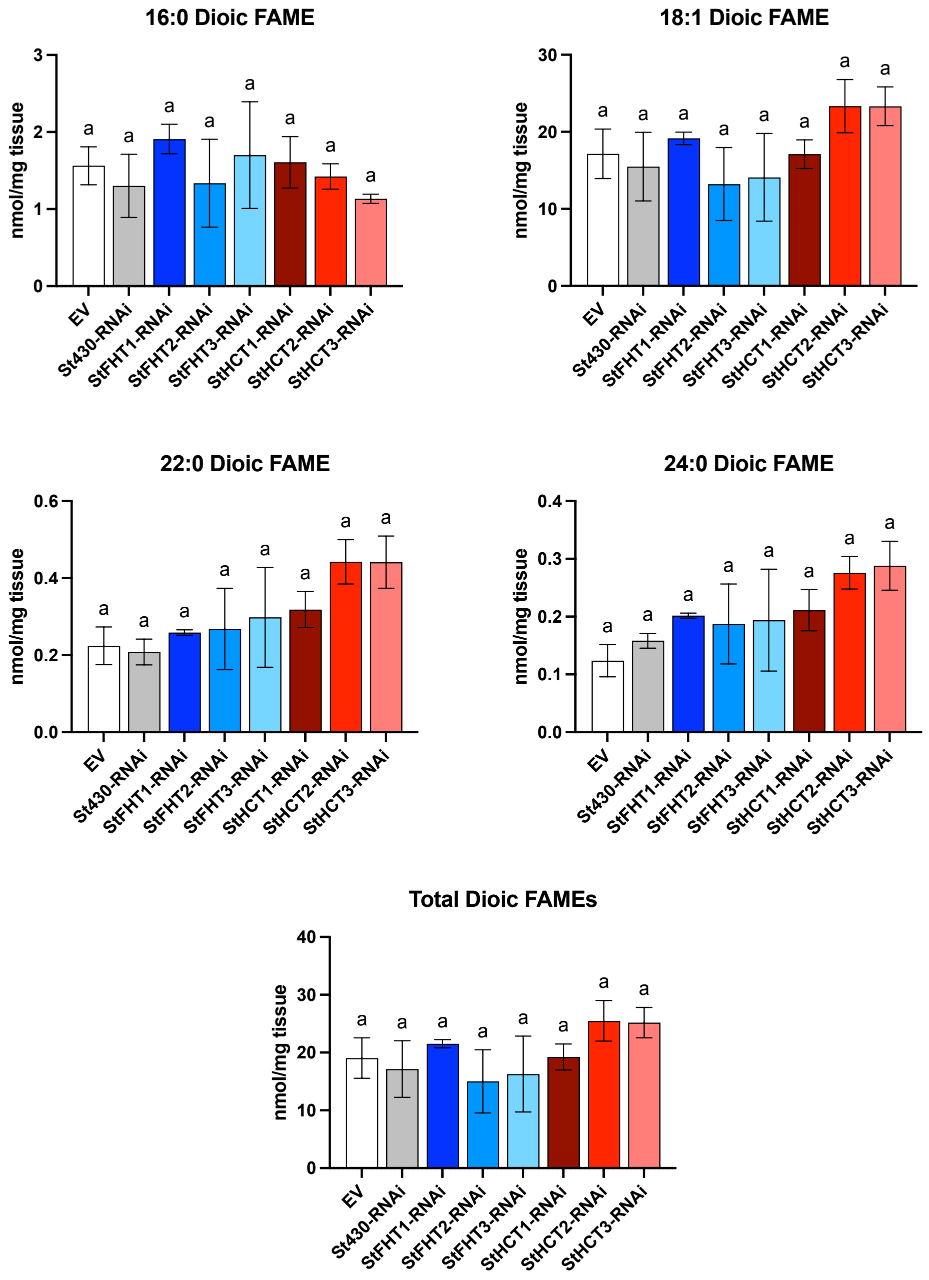

Figure S2 Poly(aliphatic) Suberin Monomers in Potato RNAi-knockdown Line Wound Periderm. Insoluble aliphatic suberin monomers were derived from extractive-free wound periderm (7 pdw) by methanolic HCL followed by trimethylsilylation and analyzed by GC-FID. Monomer chain-length distribution is shown for (A) FAMEs, (B) 1-Alkanols, (C) w-OH FAMEs and (D) a,w-Dioic FAMEs. Data for five independent EV lines were pooled (n=5). For At1g24430 homolog Soltu_DM_05G020490 (St430), three replicates from one independent RNAi line were analyzed (n=3), while for StFHT-RNAi and StHCT-RNAi lines, three replicates from each of three independent lines were analyzed (n=3).Data within each sub-panel were analyzed by One-Way ANOVA followed by Holm-Šídák's multiple comparisons test. Different letters above bars indicate significant difference (P = 0.05).

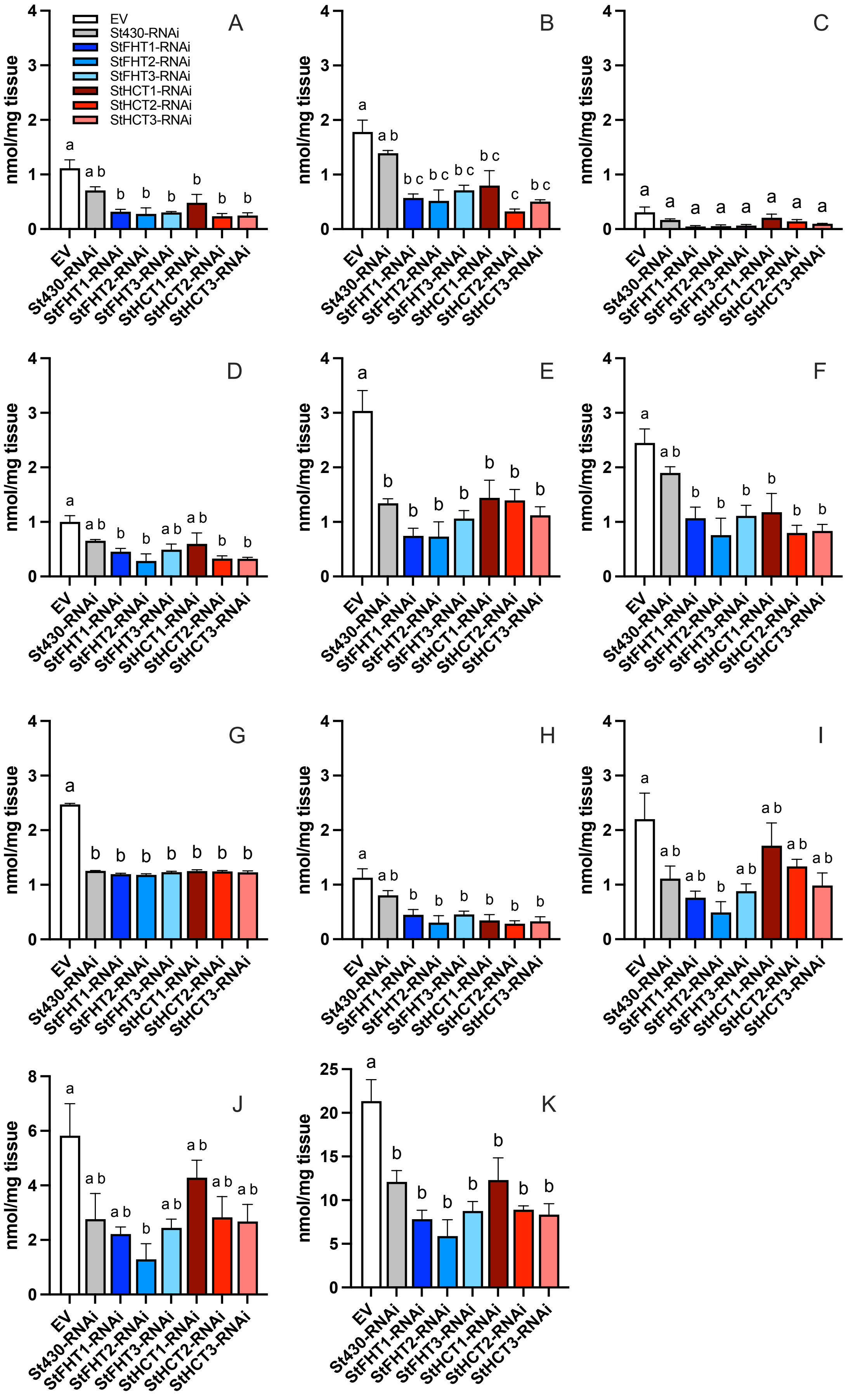
EV
St430-RNAi
StFHT1-RNAi
StFHT2-RNAi
StFHT3-RNAi
StHCT1-RNAi
StHCT2-RNAi
StHCT3-RNAi
A
B
C
D
E
F
G
H
I
J
K
nmol/mg tissue

Figure S3 Soluble Alkyl Ferulates in Potato RNAi-knockdown Line Wound Periderm.
Ferulate esters were extracted from wound periderm collected 7 dpw and analyzed by LCMS. Data are plotted by individual 1-alkanol l chain lengths. (A) 16-C, (B) 18-C, (C) 19-C, (D) 20-C, (E) 21-C, (F) 22-C, (G) 23-C, (H) 24-C, (I) 26-C, (J) 28-C and (K) Total ferulates. Data for five independent EV lines were pooled (n=5). For At1g24430 homolog Soltu_DM_05G020490 (St430), three replicates from one independent RNAi line were analyzed (n=3), while for StFHT-RNAi and StHCT-RNAi lines, three replicates from each of three independent lines were analyzed (n=3). Data were analyzed by One-Way ANOVA followed by Holm-Šídák's multiple comparisons test. Different letters above bars indicate significant difference (P = 0.05).

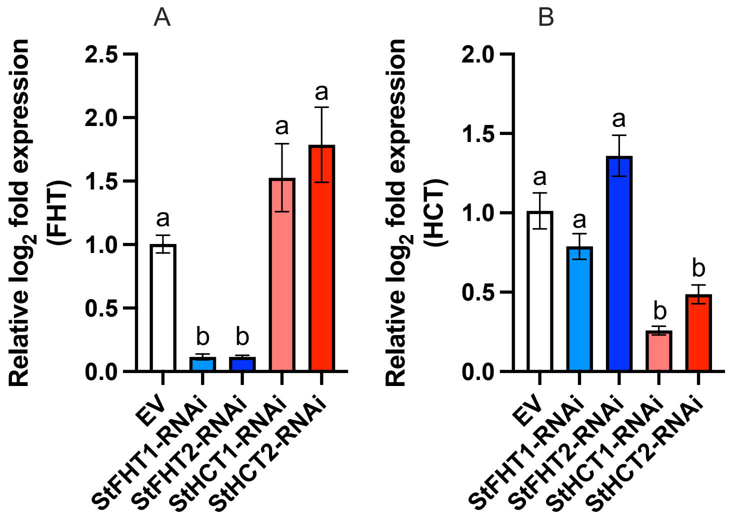

Figure S4 Acyltransferase gene expression in Potato RNAi-knockdown Line Wound Periderm.
Reciprocal qRT-PCR was performed on each of two StFHT- and StHCT-RNAi knockdown lines. (A) expression of *StFHT* in empty vector (EV), StFHT-RNAi and StHCT-RNAi lines of potato, 72 hpw. (B) expression of *StHCT* in empty vector (EV), StFHT-RNAi and StHCT-RNAi lines of potato, 72 hpw. Data within each panel were analyzed separately by One-Way ANOVA followed by Holm-Šídák's multiple comparisons test. Different letters above bars indicate significant difference (P = 0.05).

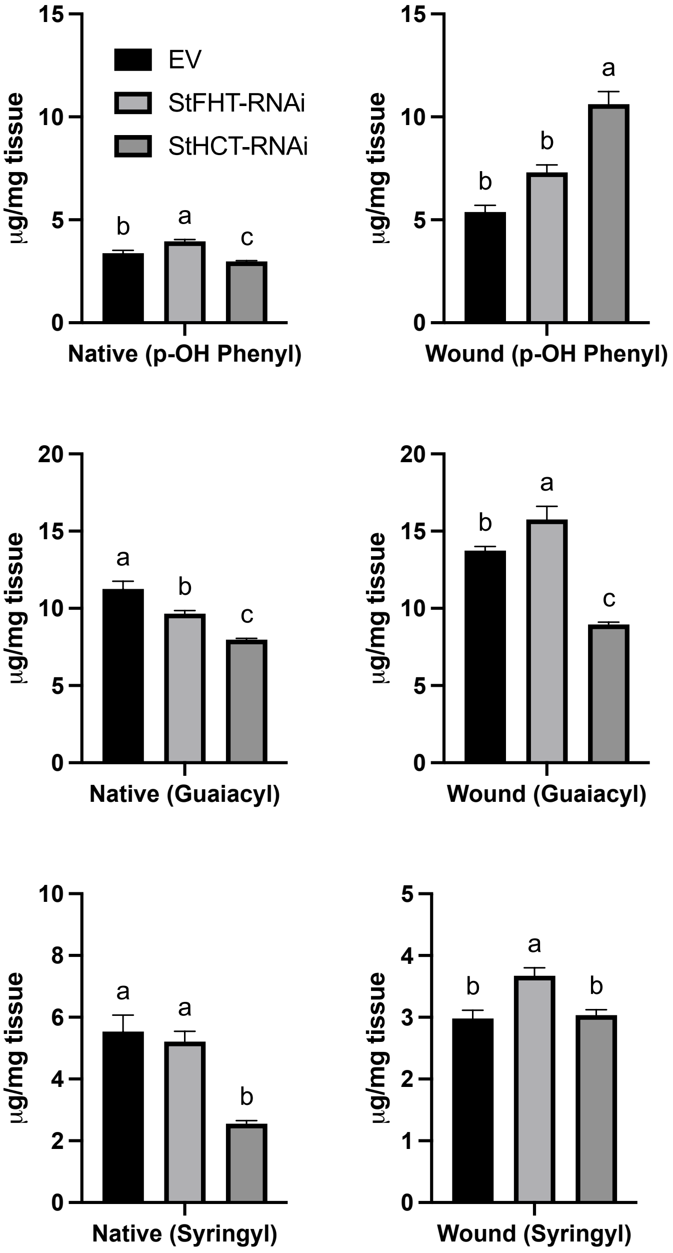

Figure S5. mNBO Monomer Comparison Across Genotypes

Monomers derived from mNBO analysis of native (left column) and wound (right column) periderm from EV, StFHT-RNAi and StHCT-RNAi lines are plotted by class. There were no statistical differences in the amount of phenolic monomers between independent lines within EV, StFHT-RNAi and StHCT-RNAi. Native and wound periderm for five independent EV lines were pooled and analyzed. For StFHT-RNAi lines, n=7 for wound-periderm and n = 9 for native-periderm, while for StHCT-RNAi lines, n=14 for native- and wound-periderm. Data were analyzed by One-Way ANOVA followed by Holm-Šídák's multiple comparisons test. Different letters above bars indicate significant difference (P = 0.05).

```
DMT400081182    --CCACACTCACAAGTATACCATTTCTAG--ACTCAAAATATCAGAACATTAACTGCATT
DMT400036695    CAACCCACTCTCAACCATATTATCACCCATAACTTAAAGGCAAAAAGCTCAACAACATT
DMT400018478    ------------------------------------------------------------

DMT400081182    TTTTCCCCGTCTTTTAGGATTTTTAATTTGAGAAATGGAGAGTGGTAAACACAATGTTGC
DMT400036695    ATCCTGCTCCTTCTC----TTTTCA---CAAGAAAACAGAGTTGATTGAT-CAACGATGA
DMT400018478    ------------------------------------------------------------

DMT400081182    AATTGAGCTTATTGTGAAGCAAGGAGTGCCAAGTTTGGTGGCACCAGCAGAGGAAACAGA
DMT400036695    AG--------ATCGAGGTGAAAAAATCAACGATGGTGCAGCCAGCGACGGAGACGCCGCA
DMT400018478    ----------ATGGAGTTGATTAAACC--TGTTTCTCCTACTCCTAATCACCTTAAATGT
                          ** * *  *     *          *

DMT400081182    GAAGGGGCAATATTACT-TATCAAATCTTGATCAGAATATAGCCGTGCCGGTTCGAACAA
DMT400036695    ACTGAGGCTGTGGAACTCTAATGTAGATTTGGTGGTGCCTAATTTCCACACGCCCAGTG-
DMT400018478    TTTGAATACTCTTTTTTGGATCAAATGGCCATACCCTTATATGCTCCCTTAGCTCTATTT
                   *            *  *    *              **

DMT400081182    TTTACTGTTTTAAATCAGAAGAAAAGGGGAATGAGAATGCTGCTGAAGTGATGAAGGATG
DMT400036695    TTTATTTCTATAGGCCAACGGGATCCCCAAATTTTTTCGACGGAAAAGTGCTTAAGGAGG
DMT400018478    TATCCTCCTCCTCATTCAAATTATGAGCAAGCAATCTCAATCAATTCAT-CGAGATTACT
                * *  *  *             *      *                  *     *  *

DMT400081182    CTTTGTCAAAGGTTCTTGTTCATTATTATCCACTAGCAGGGAGATTGACAATTAGCCAGG
DMT400036695    CACTAAGCAAAGCCCTAGTGCCGTTTTATCCGATGGGTGGGAGGCTGTGCAGAGATGAAG
DMT400018478    CGTTTTGAAGAAATCTCTTTCTGAAACTCTTACTCGTTATTACCCT-TTTGCTGGTCGAA
                *  *    *     **  * *            * *     *   *

DMT400081182    AAATGAAGCTTATTGTGGACTGTAGTGGAGAAGGGGCAGTTTTTGTTGAGGCAGAAGCA-
DMT400036695    ATGGCCGTATTGAGATTGATTGTAAAGGGCAAGGTGTGCTTTTTGTGGAAGCTGAGTCT-
DMT400018478    TCAAGGGCGTCTCAATAGAATGCAACGATGAGGGAGCGCCTTTTTACGAGGCATTTGCTC
                         *     * ** ** *  *   * ** *    ****   ** **     *

DMT400081182    --AATTGTAACATAGAGGATATTGGTGATAATACTAAGCCTGATCCTGTTACACTTGGCA
DMT400036695    --GATGGTGTGGTGGATGATTTTGGCGATTTTGCA----CCGACGTTG--GAGCTCCGAC
DMT400018478    ATAATTATGAGTTCGAGAACGTTCTTAGAAATCACGACTTAACCAAAGATTTCCTTCCCA
                   **  *    * **  *  **        *               *     **

DMT400081182    AACTTGTTTATGATATCCCTGGTGCTAAA---AATATACTAGAAATGCCTCCCCTGGTGG
DMT400036695    GACTTATTCCGGCAGTTGATTACTCACAA---GGAATCGAATCTTATGCTCTCTTAGTGT
DMT400018478    ATATTGTTGCTGAAGTTGGTTCTTCAGTATTCAATAATTCAACTTTGTATCCCCTGCTTG
                   ** **   *   *   *    *   *      *    *        ** * *  *

DMT400081182    CTCAGGTGACAAAGTTCAAATGTGGGGGATTTGTGCTTGGTTTGTGCATGAACCATTGCA
DMT400036695    TGCAGATAACACATTTTAAATGTGGGGGAGTTTCCCTTGGTGTGGGCATGCAACATCATG
DMT400018478    TCCAAGTCACACTTTTCAAATGTGGAGGTATGGTTATTTGTATGTGTGCTTTTCACCAAG
                  **  * ***   ** ******** **  *     ** ** ** *       **

DMT400081182    TGTTTGATGGGATCGGAGCGATGGAGTTTGTGAATTCTTGGGGTGAAACTGCTAGAGGTT
DMT400036695    CAGCAGATGGAGCTTCTGGTCTTCACTTCATCAACACATGGTCAGACATGGCTCGTGGTT
DMT400018478    TTGCTGACGGGGCCACGTTGTATGGCCTCGCCAACGCCTGGGCAATAGGTAGCTGCAACG
                     ** **                 *    **  * ***             *

DMT400081182    TACCTATCAAAGTCATTCCATTCCTAGACAGGAGCATACTCAAGCCTAGAAATCCACCAA
```

```
DMT400036695    TGGACATTACTATCCCACCTTTCATTGATCGTACCCTCCTCCGTGCTCGTGATCCACCTC
DMT400018478    AGTTACCTGAATTCGTTGCAGCTGCCAA-------ATTCTTACCACCACCTATCCCATAT
                            **    *        *        * **     *     ****

DMT400081182    AGCCTGAATATACTCATAACGAGT-TTGCTGAAATTGAAGATATATCAGACTCTACTAAA
DMT400036695    AGCCTCAGTTCCCCCACGTCGAGTACCAGCCACCTCCCACTCTCGAGGCAACAGAAGAAA
DMT400018478    GTCTCAAACGGACCAATGTTGCAC--TCTCAACTTACCAATTTCTGCCATAACCAAGAAC
                  *   *     *  *    *          *  *   *               *  **

DMT400081182    CTATACCAAGAAGAAATGCTGT-------ATAAGACCTTCTGTTTTGACCCTGA---GAA
DMT400036695    ATGCCCCGAATGCAGATACTGTTCCTGAAACGAGTGTGTCCATCTTCAAACTAACCCGCA
DMT400018478    GTGTTTCAAAATTATTTACCTTT---------GATGCCTCTACTATAGCATCG-------
                 *    * *    *  * *  *                **     *

DMT400081182    GCTTGAACAA----CTAAAAGCAAAAGCCAGAGAAGATGGCAATGTTACCAAATGCACTT
DMT400036695    ATCAAATCAATACCCTCAAAGCTAAGTCCAAAGAAGATGGAAATACCGTTAACTACAGCT
DMT400018478    --------------CTCAGGGCTAAGGCCATGAGCGA-GGATGTACCCGTGCCTACACG-
                              ** *  ** **  ***     ** **   *         * **

DMT400081182    CATTTGAAGTCCTTTCAGCTTTCGTCTGGAAAGCGCGAACTCAGGCATTACAAATGAAGC
DMT400036695    CCTATGAGATGTTGGCAGGACATGTGTGGCGCTCCACTTGTATGGCACGAGGACTCACTC
DMT400018478    -GGTTGAAGCTGTATCAGCTCTGATTTGGAAA--------TGTGTCATAACTGCTGGTGC
                    ***     *  ***      * ***           *  * **  *    *    *

DMT400081182    CTGATCAAAAGA--CAAAACTTCTCTTCGCTGTTGATGGAAGATCAAGATTTGATCCCTC
DMT400036695    AGGATCAAGAAA--CCAAGTTGTACATAGCAACTGATGGGCGTGCTAGGCTCCGACCTTC
DMT400018478    TGGTGCTGGTGCTTCAAAATTGACAAACTTTGTAAATATGCGAAGGCGATTTGTTCCTCC
                  *  *        * **  *              **    *     *  *    **  *

DMT400081182    AATTCCACAAGGATATTTTGGAAACGGAATTGTTTTAACTAATGCACTCTGCACTGCTGC
DMT400036695    TCTCCCACCAGGCTATTTTGGTAATGTGATATTCACAGCCACTCCTGTTGCTGTTGCTGG
DMT400018478    ATTGCCAGATCATTGTGTAGGAAATGTTATTGCAGTTGCCACAGCGTGTAAAGGTGAGAA
                  * ***      * * * ** ** *  **        * *   *         **

DMT400081182    AGAGATAGTCGAAAA----TCCACTTTCTGTTGCTGT-TAAGTTGGTTCAAGAAGCTGTT
DMT400036695    TGATCTCCAATCAAA----GCCTATTTGGTACGCAGC-CAGTAAAATCCATGATCAATTG
DMT400018478    CGATGATGGATCTGACTTGGCCACATTGGTCAATTGTATAAGAAAATCGTTGTCACAATT
                 **           *     **   **        *   *      *    *      *

DMT400081182    AAATTGGTTACAGACAGTTACATCAAATCTGCTATAGACTATTTTGAA---GCAACAAGA
DMT400036695    GCTATAATGGACAACGATTATTTAAGATCAGCTCTTGATTATTTGGAATT-GCAGCCTGA
DMT400018478    GAGTTCCAAATATGTGGACAAACAGAGTCGAGATGAGGCGATTTTCGCCTTTCCCTATGA
                    *              *       **       *   ****        *   **

DMT400081182    GCTAGGCCTTCT--------------------TTAACTGCT--ACTCTTCTTATTACTAC
DMT400036695    CTTGAAGGCTCTAGTCCGCGGTGCACATACATTTAAGTGCCCGAATTTAGGAATAACTAG
DMT400018478    CTTAATGGAGATAA-CTGAAGGGCTAATCCGAGGGGAGATC--GCGTTGCAAGTTAGTAG
                  *        *                                   *     * * **

DMT400081182    TTGGTCTAGGCTTTCTTTCCACACCACGGATTTCGGGTGGGGAGAGCCTAT-TGTATCAG
DMT400036695    CTGGTCTAGGCTGCCTATCCATGATGCTGATTTCGGATGGGGTAGGCCAATATTTATGGG
DMT400018478    CTTGTGT-GGCTATAAATCA--TACGTTGATTTTGGTTGGGGCAAACCGTCATGGGTTAG
                 * ** * ****     **         ***** ** *****    **    *   *  *

DMT400081182    GGCCAGTGGCTTTACCAGAGAAGGA--AGTTTCTCTGTTTCTTTCTCATGGGAAAGAGAG
DMT400036695    ACCTGGTGGTATTGCTTATGAAGGCTTAAGCTTTATATTGCCAAGTCCCATCAACGATGG
```

```
DMT400018478    TCCAAATCCAAGGATATCTACGAAT---AATGTTCGATTGATGGATTCAAAGGATTATGG
                  *   *                          *   **      *       *  *  *

DMT400081182    GAGAAGT-GTCAATGTGCTTCTTGG-ATTGCCAGCTTCTGCTATGAAGACATTTGAAGAA
DMT400036695    CAGTCA--ATCAGTTGCCATCTC---ACTGCAAGCAGAACATATGAAACTTTTCGAGAAG
DMT400018478    TGGAATAGATGTGTTGATATGTTTGAAAGATAAAGCTATTATGTCAAAATTTCAACAAGA
                  *      *   *     * *    *     *        * * **    *

DMT400081182    CT----TATGGAGATCTAAACAGCCATATTGCAGCTCTAATTTTCCATGAAATTTACTTT
DMT400036695    TTCTTGTATGACATTTGAAAGAAGCGGATTTCTTGTGCTGCTTTGATTGGGATTTACATT
DMT400018478    GATAGAGCTACAGTTGCTATCGTTTGGATCAGTCAACTAA--------------------
                        *     *   *        **

DMT400081182    -ATTTATGTGTAATTGATTGTGTTTTTTACTCTGTGGTGTTGTTATTATTAGTACTTTGT
DMT400036695    GACGGATATGTAAAAGAGACTCCTTTTTATCTTTT--TTTCATTAGAGGTTGTTCTTT--
DMT400018478    ------------------------------------------------------------


DMT400081182    ATTGTTATTTCTGTTTGGCATCAATTCATGCTCTTTTGCTTGTTTCTAAATGT-TCATTT
DMT400036695    ATTTCTTTTTTTGTTTGCCTGGACATCA-GTTTTATTTCTGATTCTATGATGCATTGATT
DMT400018478    ------------------------------------------------------------


DMT400081182    TTATCTCCA---TCCCATTGAGATCA----------------------------------
DMT400036695    ATATACTCAAAGTTATACTGCAAGTGTTGTATGTCTGTGAAGGACCTCAATCTTTTTTCA
DMT400018478    ------------------------------------------------------------


DMT400081182    ------------------------------------------------------------
DMT400036695    CTTCCACTAATGTGGCAAGATGTTAATGCAGAAGCTCCTAGTAGGATTGTAGCAATAGAT
DMT400018478    ------------------------------------------------------------


DMT400081182    ------------------------------------------------------------
DMT400036695    TGGAACCGCGGATGGCTTAGTTGGACTATCTTTTATTCGCTAATATTCCTGAATATCTGC
DMT400018478    ------------------------------------------------------------

                                      Legend
DMT400081182    ---------------------  selected region for StFHT
DMT400036695    AAATATAATATTATCACTGTG  selected region for StHCT
DMT400018478    ---------------------  selected region for St430
```

Figure S6. Mutiple Sequence Alignment of Potato Acytransferases *StFHT*, *StHCT* **and *St430*.**

Nucleotide sequences for *StFHT* (DMT400081182), *StHCT* (DMT400036695)and *St430* (DMT400018478) were aligned using DNAman to help select regions for RNAi targeting. The selected regions are highlighted. Dashes indicate gaps introduced by DNAman; asterisks denote conserved regions.

.
